# A Rare Case of Obstructive Uropathy in an Elderly Male From Rural India - A Case Report

**DOI:** 10.7759/cureus.42590

**Published:** 2023-07-28

**Authors:** Anshul Sood, Gaurav V Mishra, Shreya Khandelwal, Keyur Saboo, Manasa Suryadevara

**Affiliations:** 1 Radiodiagnosis, Datta Meghe Institution of Higher Education and Research, Wardha, IND; 2 Medicine, Datta Meghe Institution of Higher Education and Research, Wardha, IND

**Keywords:** elder, radiology, invagination, calculus, ureterocele

## Abstract

Ureterocele is a rare congenital anomaly often believed to be caused due to incomplete dissolution of the Chwalla membrane. In this pathology, the distal end of the ureter is invaginated in the bladder and is dilated. We present a case of an 81-year-old male from rural India who came with complaints of hematuria and was diagnosed with ureterocele.

## Introduction

Ureterocele is considered a congenital anomaly characterized by abnormal intravesical dilatation of the distal end of the ureter [1]. It can be detected prenatally or remain undetected and asymptomatic until later. The most common presenting complaint of the patient is flank pain, primarily due to the development of calculus due to urinary retention. Ureterocele might lead to the development of obstructive uropathy, which is a collective encompassing any cause of acquired or congenital, partial or complete, and permanent or intermittent obstruction of the urinary tract. Obstructive uropathy might cause permanent change in both the ureter proximal to the obstruction and renal parenchyma, which eventually drains into the abnormal collecting system [2].

## Case presentation

An 81-year-old male came to the urology outpatient department complaining of right-sided flank pain radiating to his back for ten months, which increased in severity so that the patient could not sleep at night. He also complained of on-and-off fever, burning micturition, hematuria, incomplete voiding after micturition, and incomplete clearance of the urinary bladder after micturating. There was no history of any systemic illness.

**Figure 1:**
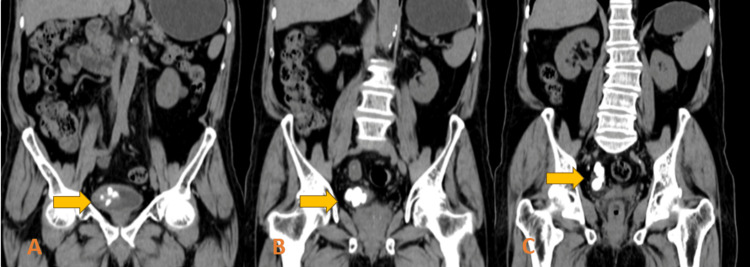
Coronal on-contrast CT abdomen showing multiple hyperdense calculi in the invaginated part of the right ureter in the urinary bladder i.e. in the ureterocele (A); in the vesico-ureteric junction (B); and in the distal ureter (C)

**Figure 2:**
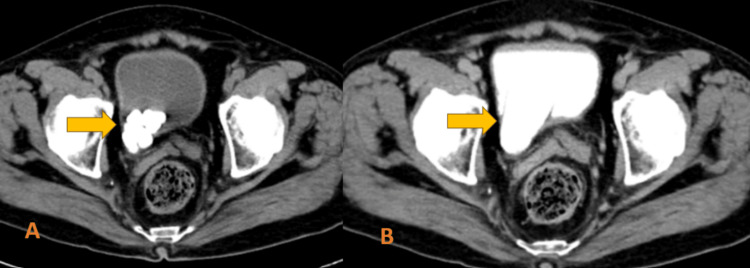
Axial CT sections of the lower abdomen non-contrast (A); and delayed phase of urography (B) showing multiple hyperdense calculi in the vesico-ureteric junction on the right side with invagination of the distal ureter into the urinary bladder

The patient has been advised ultrasonography of the abdomen and pelvis, which revealed a small calculus in the upper pole of the right kidney with dilatation of the pelvicalyceal system and visualized proximal ureter, multiple calcifications in the distal ureter and the urinary bladder. The patient was further advised to undergo a computed tomography urography scan of the kidney-ureter-bladder, which revealed a non-obstructive calculus in the upper pole of the right kidney, intravesical ureterocele with multiple calculi within and in the distal ureter. There was associated upstream dilatation of the right ureter and pelvicalyceal system, as shown in Figure 1 and Figure 2.

The patient underwent cystolitholapaxy the next day for calculi removal, and the intravesical ureterocele was reconstructed. The patient was discharged and advised to follow up with ultrasonography six months later and with micturating cystourethrography 12 months later to check for vesicoureteric reflux.

## Discussion

Ureterocele is a congenital anomaly referring to abnormal dilatation of the distal end of the ureter. The pathogenesis of the development of ureterocele is poorly understood; however, many mechanisms have been proposed, with incomplete dissolution of the Chwalla membrane being the most common [1].

These can be classified based on location as simple/orthotopic/intra-vesical when the location is at the bladder trigone or ectopic/extra-vesical when it is present in the posterior urethra or the neck of the bladder. Based on the collecting system, a ureterocele can be classified as a single-system ureterocele having a functioning kidney with a single ureter; or a duplex system having ureteric and renal duplication [3,4].

Ureterocele is managed surgically by transurethral incision of the ureterocele, where the ureterocele is removed via a urinary bladder approach [5]. Another management option includes placing the stent and removing the obstruction [6].

Unilateral cases of ureterocele account for approximately 80% and may prolapse into the bladder neck, obstructing the complete renal tract and bladder outlet obstruction [7]. Due to bladder outlet obstruction, there is the retention of urine which acts as a medium for the growth of micro-organisms and calculus.

In our case, the patient complained of incomplete voiding of the urinary bladder, which acted as a medium for the growth of multiple calculi within the ureterocele.

Uric acid calculus is the most common calculus in adults due to urine retention. Calcium phosphate, calcium oxalate, cystine, ammonium urate, and calcium-ammonium-phosphate (also known as struvite or triple phosphate calculus) are some other elements leading to calculus formation. Patients prone to urinary infections and chronic bacteriuria tend to develop calcium phosphate and struvite calculus [8].

Treatment of the calculus includes medical or surgical treatment. Medical management facilitates in the passage of ureteric calculus and stone fragments from shock wave lithotripsy [9]. Medical management may include alpha-blockers, calcium channel blockers, or steroids [10]. Surgical treatment is recommended when ureteric calculus is more than 7 mm in diameter, calculus may cause infection, or pain relief is not achieved. Surgical management may include extracorporeal shock wave lithotripsy, percutaneous nephrolithotomy, ureterorenoscopy, cystolitholaplaxy, or open surgery [11].

Nephrolithiasis or obstructive urolithiasis increases the risk for the development of chronic kidney disease, and hence dietary modification and fluid management should be encouraged among the population with the help of the government and through local programs [12]. Another complication of this pathology includes acute pyelonephritis. Management depends on the severity of symptoms and complications and also includes surgical intervention by uretero-nephrectomy [13].

Imaging modalities like ultrasound, anterograde pyelography, intravenous urography, computed tomography, and magnetic resonance imaging are used to make the diagnosis of the diseases of the urinary system and its anomalies [14].

## Conclusions

Ureterocele is a congenital anomaly and should be diagnosed antenatally. The mainstay of treatment is by surgical approach. The mother must take routine antenatal scans to check for any anomalies. Post-surgical complications are reported in cases of operated ureterocele; therefore, follow-up is very important in such patients.

## References

[1] Shokeir AA, Nijman R (2002). Ureterocele: an ongoing challenge in infancy and childhood. BJU Int.

[2] (2023). Obstructive uropathy. https://radiopaedia.org/articles/obstructive-uropathy.

[3] Gaillard F (2023). Ureterocele. https://radiopaedia.org/articles/ureterocele-1?lang=us.

[4] (2023). Ureterocele. https://www.mountsinai.org/health-library/diseases-conditions/ureterocele.

[5] Atta ON, Alhawari HH, Murshidi MM, Tarawneh E, Murshidi MM (2018). An adult ureterocele complicated by a large stone: a case report. Int J Surg Case Rep.

[6] Merlini E, Lelli Chiesa P (2004). Obstructive ureterocele-an ongoing challenge. World J Urol.

[7] (2023). Ureterocele: symptoms, causes, diagnosis, treatment. https://www.urologyhealth.org/urology-a-z/u/ureterocele.

[8] Leslie SW, Sajjad H, Murphy PB (2022). Bladder Stones. http://www.ncbi.nlm.nih.gov/books/NBK441944/.

[9] Preminger GM, Tiselius HG, Assimos DG (2023). 2007 guideline for the management of ureteral calculi. J Urol.

[10] Barnela SR, Soni SS, Saboo SS (2012). Medical management of renal stone. Indian J Endocrinol Metab.

[11] Bartoletti R, Cai T (2008). Surgical approach to urolithiasis: the state of art. Clin Cases Miner Bone Metab.

[12] Trivedi A, Kumar S (2023). Chronic kidney disease of unknown origin: think beyond common etiologies. Cureus.

[13] Nimkar SV, Yelne P, Gaidhane SA, Acharya S, Kumar S, Batra N (2022). Supernumerary kidney (triple kidney) with horseshoe malformation: a case report. Cureus.

[14] Jaiswal P, Talwar D, Acharya S, Shukla S, Kumar S (2021). Crossed fused renal ectopia with acute pyelonephritis: a case report. Cureus.

